# New York Letter

**Published:** 1892-08

**Authors:** Wm. L. Russell

**Affiliations:** 151 East 50th St. New York


### NEW YORK LETTER.

NEW YORK, July 12, 1892.

A rather ingenious apparatus has been devised for use in the treatment of that obscure and intractable disease known as writer's cramp, or grapho-spasmus, by Dr. R. Harcourt Anderson, one of the physicians to Demilt Dispensary. Dr. Anderson exhibited the apparatus at the last meeting of the County Association, and also read a short paper describing some of the more salient features of the disease. He stated that the case for which he had devised the apparatus was of seven years' duration. The patient was a clerk in a dry goods store, an occupation which required the constant use of the affected muscles in folding goods. He was incapacitated, and had received the most approved treatment from distinguished specialists. Believing that further treatment was useless Dr. Anderson attempted to overcome the muscular spasm and inco-ordination by mechanical device. After three months of experimenting he at last succeeded with the instrument in question. It consists of five soft leather bands which encircle the arm, two above the elbow and three below. The uppermost band passes around about the insertion of the deltoid, the lowermost being at the wrist. All are about one inch wide. A strong strip of rubber two inches wide passes along the anterior surface of the arm and is attached to all the bands. This rubber is lax where the forearm is flexed, but becomes tense as extension is attempted. To overcome the spasm of the flexors, there is a narrow band of calf-skin passing around the hand just back of the knuckles which is secured in the palm by a clasp. To this, on the dorsal surface, a narrow leathern strap is attached which becomes continuous with a narrow strip of rubber which passes up and is attached to the band encircling the arm just below the elbow. This rubber band is lax when the carpus is extended to its fullest extent but becomes sufficiently tense on flexion to overcome the spasmodic muscular movements. This simple device has been of the utmost value to the patient, as it enables him to write and execute other small movements which were formerly impossible.

### ETIOLOGY OF MENINGITIS.

A paper bearing on this subject was read at the last meeting of the Section on General Medicine by Dr. H. M. Biggs. He stated that he had made a bacteriological study of twenty-one cases, all of which had occurred within a month, during which the disease was unusually prevalent. Two of the cases were tubercular and two syphilitic. Of the remainder two only were secondary to otitis media. Fourteen were cerebral only and three cerebro-spinal. The effusion was purulent or sero-purulent in ten cases, and from these the investigations were principally made. The conclusion at which he had arrived was that no special organism could be regarded as the cause of the disease. The pneumococcus of Friedländer was found more frequently than any other single organism, and could be regarded as a very prevalent cause. The anthrax bacillus was found in one case in which the disease was secondary to a cellulitis of the arm in a wool-sorter. The bacillus communis coli which had been regarded as non-pathogenic was found in some cases in which it seemed to be the causative factor in the production of the disease. In all cases in which the effusion was purulent or sero-purulent, the disease was found to be microbic, whether the lesions were cerebral only or cerebro-spinal. It was found too that in cases secondary to other diseases a microbe, different from that of the original disease, might have been the cause of the meningitis.

During the discussion much interesting information was given regarding recent discoveries and theories relating to the action and reaction of pathogenic microbes and the tissues. It was stated, that just as in some epidemics of scarlet fever renal complications were common, so meningitis might become prevalent, when circumstances rendered the organism more vulnerable to the onset of various germs. The necessary conditions were still unknown, but they were probably atmospheric, as it was certain that cerebro-spinal meningitis was more prevalent in the spring. The constitutional symptoms observed were principally the effect of a toxic albumen (ptomaïne) generated by the microbes. It was believed that a substance known as anti-toxine was also formed which tended to neutralize the action of the toxine. When the organism affected this neutralization quickly it was rendered immense. Localization of the poison resulted in certain cases, as in abscess. The presence of germs which did not produce definite disease, might yet become a contributory cause by weakening the organism sufficiently to allow the development of diseased germs. The occurrence of epidemics was perhaps due to certain conditions, allowing the multiplication of microbes without the body, or rendering the organism more vulnerable.

Dr. J. D. Bryant referred to the possibility of relieving cerebral pressure in acute meningitis by operative procedures for opening the arachnoid cavity. He thought that when cerebral œdema had occurred or when effusion was into the meshes of the pia mater an operation was useless. Even when the effusion was in the sub-dural or arachnoid spaces, incision into the dura could hardly be of much service, as the opening was quickly plugged by protruding brain matter. If, too, temporary relief was obtained, the operation would more than counterbalance any possible benefit.

Dr. R. H. Sayre said that records of two cases of acute meningitis had been published, in which the removal of a small quantity of fluid had been followed by recovery.

### SANITARY NOTES.

The establishment of public baths in the crowded tenement districts of the city promises to be a useful sanitary measure.

The principal bath house is located on Center Market Place, near one of the most crowded and filthiest quarters of the city. Nine thousand baths were taken there during the month of June. The building is of brick, of pleasing appearance, and well equipped with all necessary appliances. There is an engine house, boilers and laundry in the basement; above which are the baths. These consist of a number of stalls or closets, lined and floored with stone, and divided into two compartments, which are separated by a rubber curtain. The bather disrobes in the first compartment and then passes behind the curtain, where he finds a stool and a rain or shower bath, into which he can turn the water at any temperature. Similar baths are attached to Demilt Dispensary. It might be supposed that with the many dispensaries, the sterilized milk depots, the fresh air parties and excursions for children, and now the public baths for the poor, a distinct impression would be made on the death record. This is not the case, however, for on one day last week the largest number of deaths were recorded for any single day during three years.

The splendid efficiency of the Board of Health, a matter in which the whole country has a deep interest, was sufficiently proved during the outbreak of typhus fever last winter. When it was announced in the morning papers that forty cases had been discovered simultaneously, it seemed as though a serious epidemic was inevitable. Within a week however, the matter was under control. We still hear of an occasional case but there is no thought of an epidemic. It is to be regretted that there are indications that the blighting hand of partisan politics threatens to grasp this department of the city government, and that a number of distinguished physicians who have acted as a consulting board have felt obliged to resign on that account. Dr. Janeway was the first to go, and Dr. Jacobi, Dr. Prudden, Dr. Stephen Smith, Dr. O'Dwyer, Dr. Stimson and Dr. Derby soon followed. It would be unfortunate for New York, and for the whole country, if the Health Board should lose in any degree the efficiency and energy which have characterized it in the past.

WM. L. RUSSELL.

151 *East 50th St.*

